# Changes of clinical characteristics, distribution of red flags and prognosis in contemporary patients with wild-type transthyretin amyloidosis cardiomyopathy

**DOI:** 10.1080/07853890.2024.2398735

**Published:** 2024-09-09

**Authors:** Larsen Sanne Bøjet, Ladefoged Bertil, Pedersen Anders Lehmann Dahl, Skov Jens Kæstel, Clemmensen Tor Skibsted, Poulsen Steen Hvitfeldt

**Affiliations:** Department of Cardiology, Aarhus University Hospital, Aarhus N, Denmark

**Keywords:** Cardiac amyloidosis, heart failure, survival, NT-proBNP, red flags, prognosis

## Abstract

**Aim:**

Increased diagnostic awareness and specific disease treatments have changed the landscape of transthyretin cardiac amyloidosis (ATTR). Patients with wild-type ATTR (ATTRwt) are increasingly being diagnosed, potentially changing the clinical profile and prognosis compared with existing retrospective data. We aimed to study the clinical characteristics, distribution of red flags and prognosis of contemporary ATTRwt patients.

**Methods:**

From January 1^st^ 2017, to December 31^st^ 2022, 213 consecutive patients were diagnosed with ATTRwt and prospectively followed up. Data on clinical characteristics, biomarkers, echocardiography findings, hospitalization due to worsening heart failure (WHF) and all-cause mortality were collected.

**Results:**

A 37% increase in newly diagnosed patients from 2017–2019 (*n* = 90) vs. 2020–2022 (*n* = 123) was observed. The majority of patients presented with NAC disease stage I in the latter period (49% in 2017–2019 vs. 58% in 2020–2022, *p* = .16). Red flags were primarily cardiac-related, including elevated NT-proBNP, impaired left ventricular longitudinal systolic strain with an apical sparing pattern, heart failure with increased left ventricular wall thickness and elevated troponins. NAC disease stage I as well as low NT-proBNP levels (<1000 ng/L) were significantly associated with better survival (both *p* < .001). When compared with NAC disease stage II + III combined, patients with NAC disease stage I had a significantly lower risk of WHF hospitalization or death (log rank test: *p* = .0001). Independent predictors of the combined endpoint WHF hospitalization or death were NT-proBNP (HR 1.03 [95% CI 1.00–1.07], *p* < .049) and prior implantation of permanent pacemaker (HR 2.01 [1.30–3.11], *p* = .002).

**Conclusion:**

Increased diagnostic awareness resulted in a 37% increase in newly diagnosed patients in 2020–2022 vs. 2017–2019. As expected all-cause mortality but also the morbidity in terms of risk of hospitalization with WHF were significantly lower in patients with NAC disease stage I, as well as in those with low NT-proBNP levels <1000 ng/L. These findings underline the importance of continuous attention to diagnostic awareness, as early diagnosis is critical for initiating both general and specific ATTR treatment, thus improving prognosis.

## Introduction

Wild-type transthyretin cardiac amyloidosis (ATTRwt) is a progressive and infiltrative cardiomyopathy caused by the extracellular deposition of transthyretin-derived insoluble amyloid fibrils, resulting in myocardial thickening and restrictive cardiac function [1,2]. Historically, ATTRwt has been severely under-recognized as a cause of heart failure among elderly persons who have often been misdiagnosed [1]. The clinical presentation of ATTRwt is often influenced by other coexisting cardiac disorders, leading to phenotypic heterogeneity. In descriptive studies, up to 35% of patients have previously been misdiagnosed with other cardiovascular conditions, including hypertensive cardiomyopathy, hypertrophic cardiomyopathy, ischemic heart disease, heart failure with preserved ejection fraction and aortic valve stenosis [2].

In recent years, increased disease awareness, introduction of new disease-specific treatments, use of diagnostic red flags and introduction of widely available noninvasive bone scintigraphy have resulted in a dramatic increase in ATTRwt diagnoses among elderly patients presenting with heart failure [3]. Prognosis can be estimated using the National Amyloidosis Centre (NAC) ATTR disease staging system [4,5]. However, early diagnosis is critical to initiate proper general and specific ATTR treatment, thus improving prognosis. The rapidly increasing number of scientific publications reflects the growing interest in ATTRwt [6]. However, the vast majority of ATTRwt publications include patients diagnosed decades ago, and it is likely that these patients differ both clinically and prognostically compared to patients diagnosed today [3,7–9]. Recent data suggest that more than 50% of ATTR patients are now diagnosed in the early disease stage of NAC I [3,10–12]. However, both clinical and prognostic data in recently diagnosed ATTRwt patients, as well as the diagnostic temporal influence of these data, are limited.

Therefore, we aimed primarily to study the clinical characteristics, distribution of diagnostic red flags and prognosis with respect to hospitalization due to worsening heart failure (WHF) and all-cause mortality in a large ATTRwt patient cohort diagnosed from 2017 to 2022. The temporal diagnostic influence on the clinical and prognostic characteristics was also studied, as we compared patients diagnosed in the first period (2017–2019) with patients diagnosed in the latest period (2020–2022).

## Methods

We included 213 consecutive patients diagnosed with ATTRwt at Aarhus University Hospital, Denmark, from 1 January 2017 to 31 December 2022. Patient data were collected in a dedicated prospective database established alongside the introduction of routine use of bone-scintigraphy for the diagnosis of ATTR at the end of 2016, including ATTRwt patients diagnosed and followed at Aarhus University Hospital. Data extraction included all clinical, biochemical, imaging and pathological data, hospitalization due to heart failure and all-cause mortality. To study the temporal diagnostic influence on the clinical and prognostic characteristics, we compared ATTRwt patients diagnosed in the time interval 2017–2019 with patients diagnosed in 2020–2022.

All patients underwent a comprehensive echocardiographic examination, blood sampling for biomarker analysis and had electrocardiograms (ECGs) taken. Patients were prospectively followed until the date of death, hospitalization due to WHF, or the censoring date of 20 March 2023. Follow-up was performed using data collected from either the regional database *The Electronic Patient Journal* or the national database *The Health Journal* providing the investigators with admission data from across Denmark. Hospitalization due to WHF was defined as the development or worsening of existing symptoms and/or clinical signs of heart failure requiring non-scheduled inpatient hospitalization with treatment with intravenous diuretics, pleural or ascites drainage, inotropic therapy, or dialysis initiation. In addition, WHF should be primarily related to ATTRwt cardiomyopathy. All-cause mortality data were obtained from the Danish Central Personal Registry. Investigators had complete access to all of the aforementioned data from the time of diagnosis and during follow-up. ATTRwt disease stage was evaluated in accordance with the NAC staging system, London, UK [4]. With the introduction of a new modified sub-classification of NAC I based on diuretic dosage use and level of NT-proBNP (< 1000 vs. 1000–2999 ng/L) [13], we also decided to investigate the prognostic impact of low NT-proBNP (< 1000 ng/L) on survival and WHF hospitalization. We used the cut-off of 1000 ng/L as we also included patients with atrial fibrillation, which is a prevalent clinical feature across all categories of ATTRwt. At the time of diagnosis, the following data were obtained: information about patient symptoms, medical history, comorbidities, medication, biochemistry (including N-terminal pro-B-type natriuretic peptide [NT-proBNP], troponin I and estimated glomerular filtration rate [eGFR]), echocardiography and ECG.

The ATTRwt diagnosis was established by either amyloid-positive endomyocardial biopsy using Congo red staining and immunohistochemistry and/or mass spectrometry, or by positive 99mTc-DPD scintigraphy with a Perugini Grade 2 or 3. Furthermore, ATTRwt diagnosis required negative serum and urine immunofixation analyses and a normal plasma kappa/lambda light chain ratio [14]. Both scintigraphy and biopsy were performed in patients with positive immunofixation analysis and/or an abnormal kappa/light chain ratio. Normal plasma kappa/lambda light chains were considered in the interval 0.26–1.65 [14]. All patients were genotyped to exclude hereditary ATTR.

A total of 61 patients were included in randomized controlled trials, thus receiving either acoramidis (Attribute trial), patisaran (Apollo B trial), vutrisaran (Helios B trial), or placebo. None of the patients received tafamidis treatment due to its lack of financial approval for clinical use in ATTRwt in Denmark.

### Echocardiography

Echocardiography was performed using the GE Vivid 9 or E95 ultrasound unit (Horten, Norway). All patients underwent a comprehensive two-dimensional echocardiographic assessment in accordance with the current guidelines [15]. Left ventricular ejection fraction (LVEF) was calculated using Simpson’s biplane method. Peak systolic left ventricular global longitudinal strain (LVGLS) magnitude was obtained using automated function imaging in standard two-dimensional cine loops with a frame rate *>*55 frames/s. The regional speckle area of interest was manually adjusted to obtain the optimal tracking results. The LVGLS was calculated using a 17-segment model at the time of systole when the value peaked. A LVGLS ≥ 18% (numeric) was considered to be within normal limits. In patients with atrial fibrillation, triplane images were obtained for the strain calculation. The relative apical sparing pattern (RAS) was assessed as a visual pattern on the LVGLS plot and calculated as the RASr, in which the mean strain of the five apical segments was divided by the mean of the 12 middle and basal segments. A RASr ≥ 1.5 was considered abnormal [16]. Left atrial volume index was assessed using the biplane method indexed to the body surface area. Data analyses were performed using the EchoPAC PC SW-Only, Version 202 (GE Healthcare, Milwaukee, WI, USA).

### Definition of red flags

Clinical, biochemical, electrocardiographic and echocardiographic data, referred to and acknowledged as red flags, were collected at baseline. The following cut-off points were used:

NT-proBNP > 300 ng/L; apical sparing pattern was defined as a RASr > 1.5; heart failure was defined according to guidelines [17]; increased troponin was defined as troponin *I* > 47 ng/L (145 of 166 measured patients) or troponin *T* > 14 ng/L (82 out of 83 measured patients, both troponin I and T were measured in 49 patients); carpal tunnel syndrome included only patients who have had surgery and positive electromyographic examination; low voltage was defined as a QRS voltage amplitude ≤ 0.5 mV in all limb leads or ≤ 1.0 mV in all precordial leads; conduction disorder was defined as first degree atrioventricular block or pacemaker due to advanced atrioventricular block; diagnosis of aortic valve stenosis independent of type or severity [14].

### Statistical analysis

Continuous data were presented as mean and standard deviation (SD) or median and interquartile range (IQR), as appropriate. Categorical data are presented as percentages (number). Q-Q plots and histograms were used to evaluate the normality of the data distribution for continuous variables. Differences between two unpaired groups were tested using a two-sided t-test or Mann-Whitney test, as appropriate. Differences between groups were calculated using one-way analysis of variance with Bonferroni corrections or the Kruskal-Wallis test for normal and skewed distributions. Survival analysis and analysis on a composite endpoint of WHF hospitalization and death was illustrated using Kaplan-Meier estimates, and differences between groups were compared using the log-rank test. Multivariate analysis was performed with significant predictors in the univariate analysis, in addition to comorbidities for adjustment. Hazard ratios, 95% confidence intervals (CI) and two-sided P values were determined using Cox proportional-hazard regression models. Statistical significance was set at *p* < .05. All data were collected from a database (Aarhus University, Research Electronic Data Capture) designed for this study and analysed using Stata/IC 16 software (StataCorp, College Station, Texas).

## Results

The baseline characteristics of all patients are shown in Table 1, along with a comparison between patients diagnosed between 2017–2019 and 2020–2022. An increasing number of patients were diagnosed with ATTRwt from 2017–2019 (*n* = 90) to 2020–2022 (*n* = 123), corresponding to a 37% increase.

**Table 1.** Patient characteristics of early and late diagnostic time period.

Variable	Total *n* = 213	2017–2019 *n* = 90	2020–2022 *n* = 123	*p* Value
Age (years), mean ± SD	82 ± 6	82 ± 6	82 ± 6	.47
Male gender, (%) (n)	90 (191)	92 (83)	88 (108)	.30
Hypertension, % (n)	63 (135)	68 (61)	60 (74)	.25
IHD, % (n)	28 (39)	22 (20)	15 (19)	.21
Prior implantation of PPM, % (n)	23 (48)	30 (27)	17 (21)	.03
First degree AVB, % (n)	18 (38)	11 (10)	23 (28)	.03
Aortic stenosis, % (n)	24 (50)	27 (24)	21 (26)	.35
Diabetes, % (n)	17 (37)	18 (16)	17 (21)	.89
Cancer, % (n)	21 (44)	16 (14)	24 (30)	.12
COPD, % (n)	13 (27)	11 (10)	14 (17)	.81
Loop diuretics, % (n)	73(155)	74 (67)	72 (88)	.64
Thiazide diuretics, % (n)	9 (19)	11 (10)	7 (9)	.34
Mineralocorticoid, % (n)	6 (12)	8 (7)	4 (5)	.25
Betablocker, % (n)	40 (86)	41 (37)	40 (49)	.85
ACE or ARB, % (n)	48 (102)	47 (42)	49 (60)	.76
Atrial fibrillation, % (n)	39 (82)	43 (39)	35 (43)	.21
CTS, % (n)	36 (76)	40 (36)	33 (40)	.26
Spinal stenosis, % (n)	9 (18)	7 (6)	10 (12)	.42
Tendon rupture %, (n)	10 (21)	12 (11)	8 (10)	.32
Ligament disorder (combined), % (n)	46 (98)	51 (46)	42 (52)	.20
Biochemistry				
NT-proBNP (ng/L), median (IQR)	2243	2465	2198	.19
	(1244–4174)	(1266–4569)	(1204–3634)	
Troponin I, ng/L	56 [30–87]	69 [41–101]	49 [28–74]	.009
eGFR (mL/min), % (n)				.31
>45 mL/min	78 (166)	83 (75)	74 (91)	
>30–≤45 mL/min	13 (28)	9 (8)	16 (20)	
≤30 mL/min	9 (18)	8 (7)	9 (11)	
NYHA, I/II/III–IV, %	28/47/27	26/49/25	29/45/26	.91
NAC I/II/III, %	55/31/1	49/37/14	59/27/15	.28
Echocardiography				
LVEF (%), mean ± SD	48 ± 11	47 ± 10	49 ± 11	.18
LVGLS(%), mean ± SD	11.3 ± 4	11.4 ± 3	11.3 ± 4	.79
LVEDV (mL), mean ± SD	92 ± 30	96 ± 30	89 ± 30	.10
LVESV (mL), mean ± SD	49 ± 21	51 ± 21	47 ± 21	.12
LVSV (mL), mean ± SD	44 ± 16	45 ± 17	43 ± 16	.42
LAVi (mL/m^2^), median (IQR)	66(44–92)	66 (45–95)	65 (37–84)	.58
IVS (mm), mean ± SD	16 ± 3	17 ± 3	16 ± 3	.27
PW (mm), mean ± SD	14 ± 3	14 ± 3	14 ± 3	.37
RWT, median [IQR]	0.6 [0.5–0.7]	0.6 [0.5–0.7]	0.6 [0.5–0.8]	.12

*Note:* ACE: angiotensin-converting enzyme; ARB: angiotensin receptor blocker; AVB: atrioventricular block; IHD: ischaemic heart disease; COPD: chronic obstructive pulmonary disease; CTS: carpal tunnel syndrome; eGFR: estimated glomerular filtration rate; LVEF: left ventricular ejection fraction; LVGLS: left ventricular global longitudinal strain; LVESV: left ventricular end-systolic volume; LVSV: left ventricular stroke volume; LAVi: left atrial volume index; IVS: interventricular septum; PPM: permanent pacemaker; PWT: posterior wall thickness; RWT: relative wall thickness.

### Clinical characteristics according to time of diagnosis (2017–2019 vs. 2020–2022)

**Figure 1.**
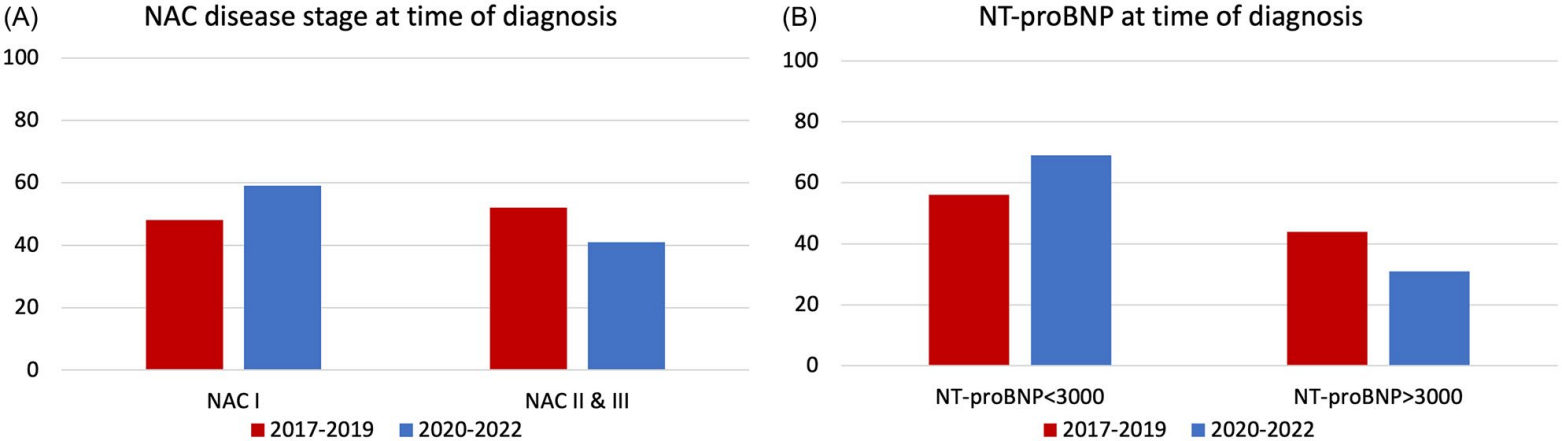
(A) Proportion of patients presenting with NAC I vs. NAC II + III at the time of ATTRwt diagnosis in 2017–2019 vs. 2020–2022. (B) Proportion of patients presenting with NT-proBNP <3000 ng/L vs. > 3000 ng/L at the time of diagnosis in 2017–2019 vs. 2020–2022.

A tendency towards a larger proportion of patients in NAC disease stage I at the time of diagnosis was observed in the period 2020–2022 as opposed to the 2017–2019 period (58% vs. 49%, *p* = .163, Figure 1(A)). Also, a borderline significant larger proportion of patients had NT-proBNP < 3000 ng/L when diagnosed with ATTRwt in 2020–2022 as compared with those diagnosed in 2017–2019 (68% vs. 57%, *p* = .082, Figure 1(B)). The proportion of patients with even lower NT-proBNP < 1000 ng/L at time of diagnosis increased in the most recent period (21% in 2020–2022 vs. 18% in 2017–2019, *p* = .54), although not statistically significant. There was no difference in the eGFR or NYHA class distribution between the two time periods (Table 1). None of the patients received SGLT2 inhibitors. Finally, no significant differences were noted with respect to left ventricular structural or functional parameters assessed by echocardiography in patients diagnosed in the early vs. late period.

### Distribution of diagnostic red flags

**Figure 2.**
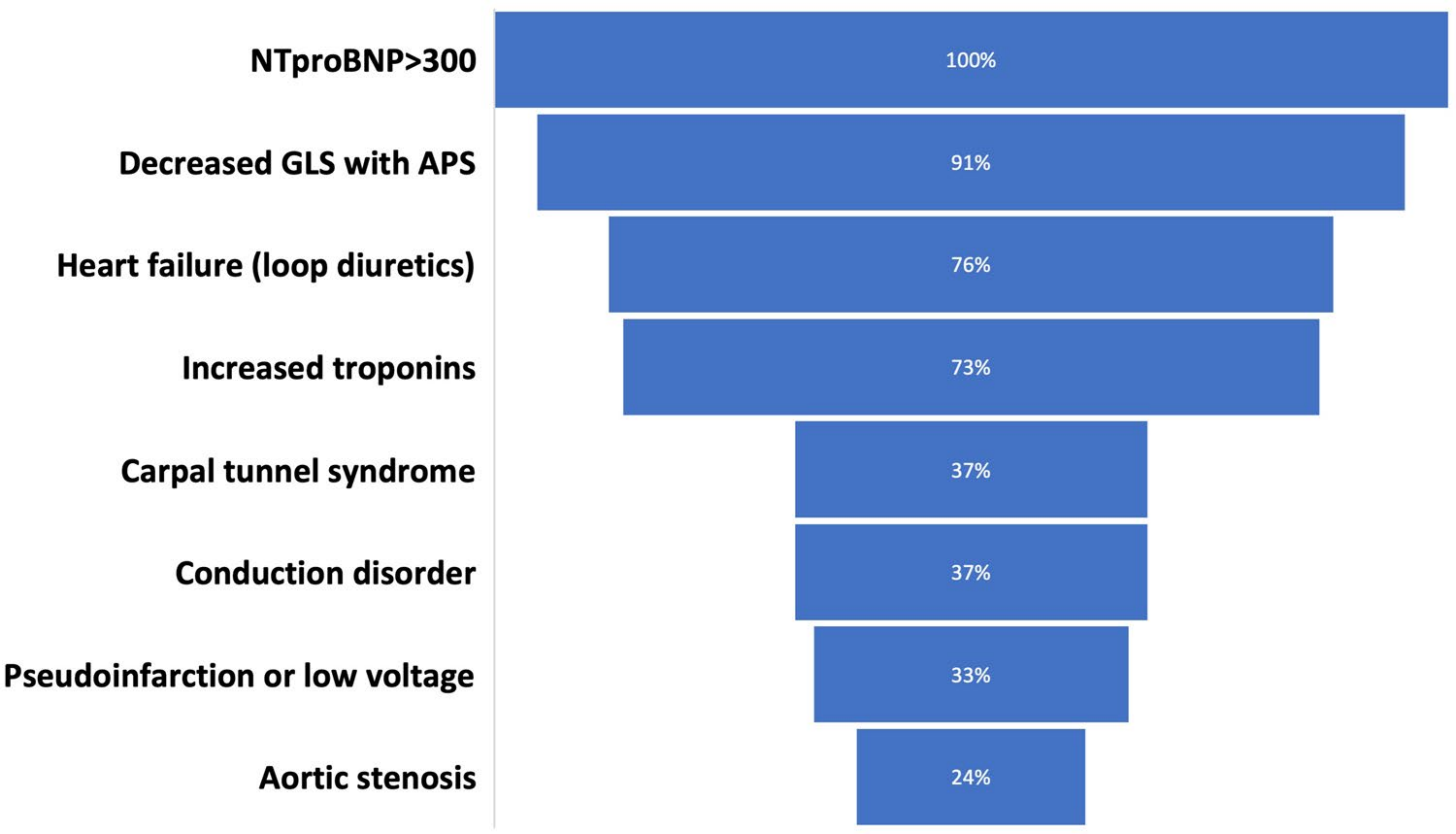
Distribution of red flags among patients with increased left ventricular wall thickness at time of ATTRwt diagnosis. All numbers are presented as percentages. APS: apical sparing pattern; conduction disorders: first degree atrioventricular block or pacemaker due to advanced atrioventricular block; GLS: global longitudinal strain; NT-proBNP: N-terminal pro-brain natriuretic peptides.

The majority of patients (*n* = 204 [96%]) presented with left ventricular wall thickening on echocardiography at the time of ATTRwt diagnosis. Of these, all 204 patients (100%) had NT-proBNP levels > 300 ng/L, 187 (91%) had reduced LVGLS (<18% numerical) with apical sparing pattern, 155 (76%) had heart failure symptoms requiring treatment with loop diuretics, (37%) had prior carpal tunnel syndrome surgery, and 50 (24%) had a diagnosis of aortic stenosis of varying type and severity. Finally, 76 (37%) of the patients presented with signs of electrical conduction disturbances with either first degree atrioventricular block or prior implantation of permanent pacemaker (PPM) due to advanced atrioventricular block. Abnormal ECG with pseudo-infarction or low voltage pattern was found in 68 (33%) patients (Figure 2). When comparing prevalence of red flags between the two time periods, significantly more patients had LVGLS with apical sparing when diagnosed in 2017–2019 as compared with 2020–2022 (97% vs. 88%, *p* = .02). Also, a higher prevalence of elevated troponin I was found in patients diagnosed in 2017–2019 as compared in 2020–2022 (68% vs. 51%, *p* = .04). There was no difference in the distribution of other red flags.

### Patient survival and risk of WHF hospitalization

The number of deaths was 65 (31%) during a median follow-up of 798 days (26 months) (IQR 397–1195 days). Estimated survival by Kaplan-Meier analysis at one year was 91% (95% CI 86–94), at two years 82% (76–87) and at three years 68% (60–75). As expected, a lower NAC disease stage was significantly associated with better survival (*p* < .0001). One-year survival in all patients according to NAC I, II and III disease stages was 97% (92–99), 85% (74–92), 79 (60–90) whereas two-year survival was 91% (83–95), 77% (64–86) and 62% (41–78), respectively. Finally, three-year survival was 83% (73–90) for NAC I, 57% (41–70) for NAC II and 38% (18–59) for NAC III. In patients with NT-proBNP <1000 ng/L, one-year survival was 98% (100 − 84), two-years survival was 95% (80–99) and three-years survival was 95% (80–99). In patients with NT-proBNP > 3000 ng/L, survival after one year was 81% (70–88), after two years 67% (55–77) and after three years 45% (31–57). Overall, there was no significant difference in survival between patients diagnosed with ATTRwt in 2017–2019 vs. 2020–2022 (*p* = .09).

The overall risk of hospitalization due to WHF was 27% (21–34) within one year, 42% (35–49) within two years and 48% (41–56) within three years after ATTRwt diagnosis. Risk of hospitalization within two years was higher in patients diagnosed in 2017–2019 vs. 2020–2022 (48% vs. 34%, *p* = .05), suggesting a trend towards improved prognosis. Stratified by NAC disease stages I, II and III, risk of hospitalization due to WHF after one year was 19% (13–28), 36 (25–49), 37% (23–57). After two years the risk was 35% (27–46); 50% (37–63); 50% (33–70) and after three years 41% (32–52); 55% (42–68); 61% (42–80).

In patients with NT-proBNP < 1000 ng/L, the risk of WHF hospitalization after one year was 13% (5–27), after two years 30% (18–48) and after three years 42% (27–62). The risk increased in patients with NT-proBNP levels > 3000 ng/L; after one year 37% (27–49), after two years 48% (37–61) and after three years 57% (45–69) (log rank test: *p* = .15).

**Figure 3.**
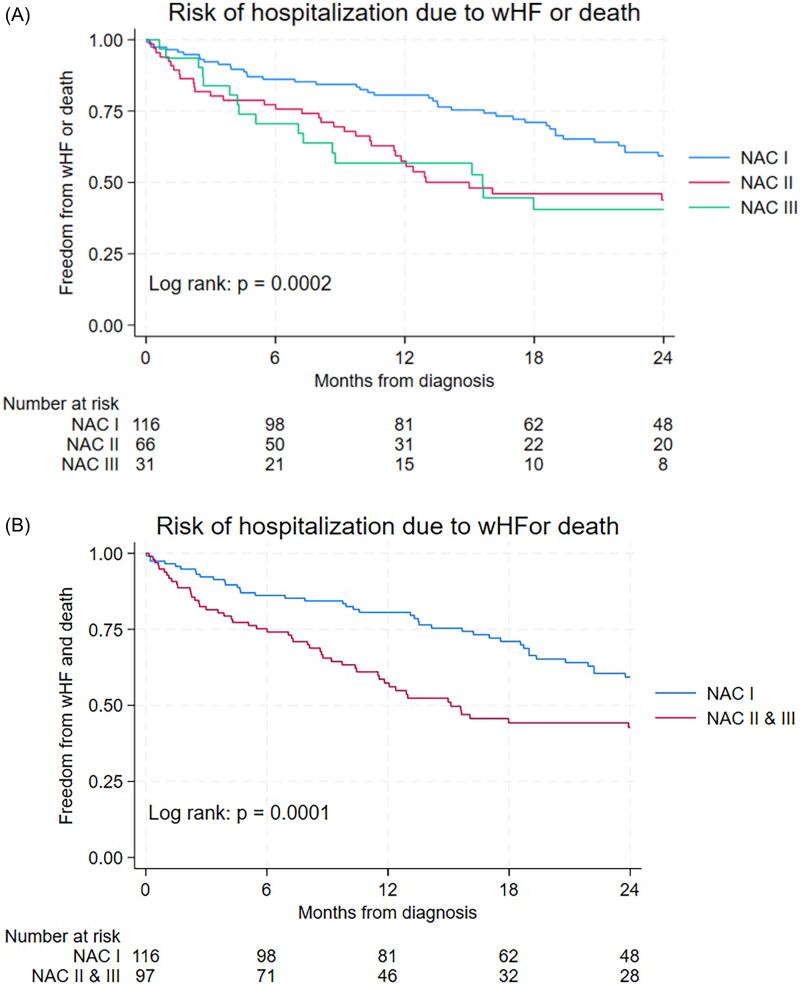
(A) Risk of hospitalization with worsening heart failure or death stratified by NAC disease stage. (B) Risk of hospitalization with worsening heart failure or death in NAC disease stage I vs. NAC disease stage II or III.

**Figure 4.**
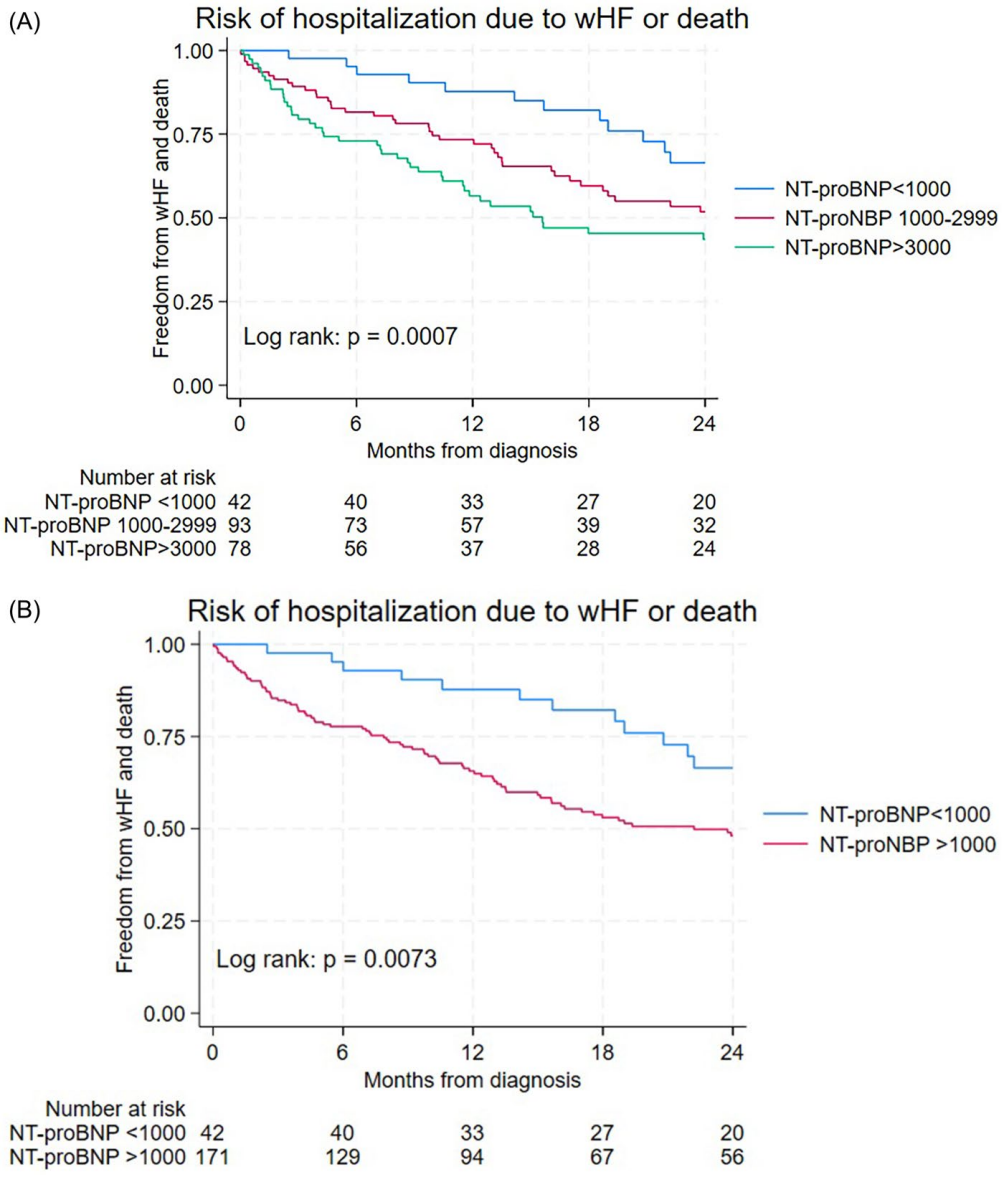
(A) Risk of hospitalization with worsening heart failure or death stratified by NT-proBNP. (B) Risk of hospitalization with worsening heart failure or death in patients with NT-proBNP <1000 ng/L vs. >1000 ng/L.

**Figure 5.**
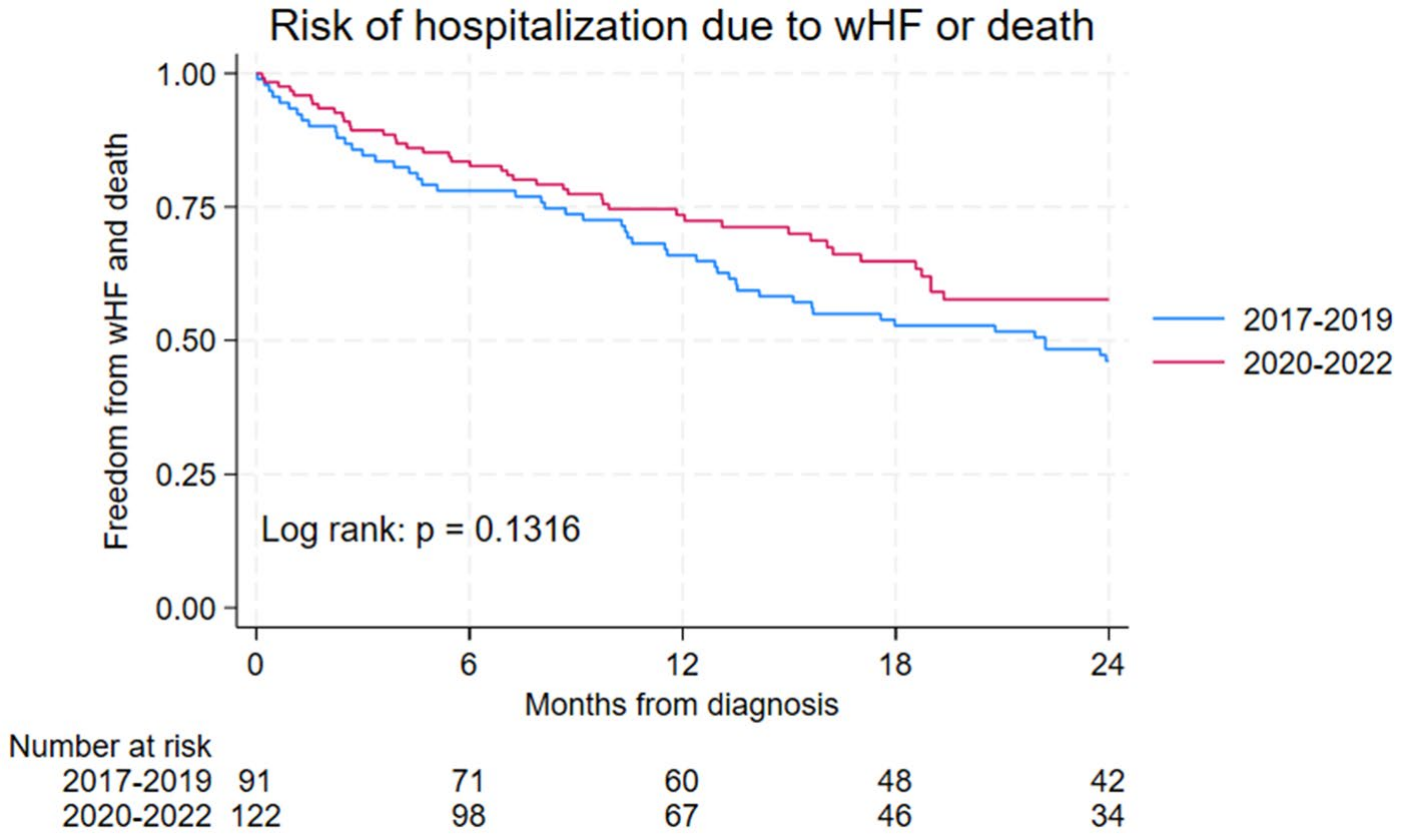
Risk of hospitalization with worsening heart failure or death in patients diagnosed with ATTRwt in 2017–2019 vs. 2020–2022.

The risk of WHF hospitalization with death as competing risk was significantly different between NAC disease stages as shown in Figure 3(A) (*p* < .0002). Patients in NAC disease stage I had significantly lower risk of WHF hospitalization or death as compared with patients in NAC disease stage II or III as demonstrated in Figure 3(B) (*p* < .0001). The risk of WHF hospitalization or death increased significantly with increasing levels of NT-proBNP (< 1000;1000–2999; ≥ 3000 ng/L, *p* = .0007) as shown in Figure 4(A). In particular, patients with low NT-proBNP (< 1000 ng/L) had significantly lower risk of WHF hospitalization or death as compared with patients having high NT-proBNP levels (*p* = .0073) as demonstrated in Figure 4(B). The risk of WHF hospitalization or death tended to be lower in patients diagnosed in 2020–2022 as compared to 2017–2019, however, not statistically significant (*p* = .13, Figure 5).

### Independent predictors of WHF hospitalization and all-cause mortality

Multivariate Cox regression analyses identified prior implantation of PPM, eGFR and NT-proBNP as independent predictors of all-cause mortality (Table 2). With regard to WHF hospitalization, prior implantation of PPM and aortic stenosis remained independent predictors of WHF admission in the adjusted multivariate analyses. Independent predictors of the composite event of WHF hospitalization or death were prior implantation of PPM and increasing NT-proBNP.

**Table 2.** Univariate and multivariate cox regression analysis of predictors of survival and hospitalization due to worsening heart failure.

	Univariate			Multivariate		
Variable	HR	*p* Value	95 % CI	HR	*p* Value	95% CI
Mortality						
Age, years	1.00	.64	1.00–1.00	1.00	.89	1.00–1.01
Sex, male	1.18	.68	0.54–2.61	1.90	.14	0.81–4.44
Diabetes	1.67	.09	0.93–2.99	1.59	.18	0.81–3.11
Hypertension	1.27	.90	0.75–2.16	0.85	.61	0.46–1.57
Aortic stenosis	1.26	.40	0.73–2.17	1.48	.18	0.84–2.63
IHD	1.09	.78	0.48–2.05	1.00	.99	0.50–2.02
Atrial fibrillation	1.31	.28	0.80–2.14			
Pacemaker	2.18	.002	1.032–3.60	2.42	.002	1.36–4.27
eGFR	0.98	.001	0.97–0.99	0.98	.01	0.97–0.99
RWT	0.88	.691	0.47–1.66			
NT-proBNP/1000	1.07	<.001	1.04–1.10	1.05	<.001	1.04–1.10
LVEF	0.97	.007	0.94–0.99			
LVGLS	0.91	.01	0.85–0.98	0.95	.18	0.87–1.03
Hospitalization due to worsening heart failure						
Age, years	1.00	.79	0.99–1.01	1.00	.86	0.98–1.02
Sex, male	0.89	.74	0.45–1.77	1.12	.77	0.52–2.42
Diabetes	1.97	.004	1.24–3.14	1.60	.09	0.93–2.76
Hypertension	1.56	.055	0.99–2.45	1.17	.54	0.70–1.95
Aortic stenosis	1.70	.017	1.10–2.63	1.77	.02	1.12–2.81
IHD	1.28	.34	0.77–2.12	1.00	.99	0.57–1.74
Atrial fibrillation	1.15	.50	0.76–1.74			
Pacemaker	2.14	.001	1.39–3.28	1.93	.006	1.21–3.10
eGFR	0.99	.086	0.98–1.00	1.00	.64	0.99–1.01
RWT	1.11	.544	0.80–1.53			
NT-proBNP/1000	1.02	.21	0.99–1.06	1.02	.29	0.98–1.07
LVEF	0.98	.015	0.96–0.99	0.98	.08	0.96–1.00
LVGLS	0.94	.06	0.89–1.00			
Composite of hospitalization due to worsening heart failure or death						
Age, years	1.03	.051	1.00–1.06	1.02	.225	0.99–1.06
Sex, male	1.15	.634	0.65–2.05	1.29	.449	0.67–2.50
Diabetes	1.70	.017	1.10–2.62	1.60	.081	0.94–2.69
Hypertension	1.32	.162	0.89–1.95	0.96	.846	0.61–1.50
Aortic stenosis	1.55	.032	1.04–2.30	1.54	.044	1.01–2.34
IHD	1.08	.749	0.68–1.72	0.86	.562	0.51–1.44
Atrial fibrillation	1.24	.253	0.86–1.80			
Pacemaker	2.08	<.001	1.40–3.06	2.01	.002	1.30–3.11
eGFR	0.96	.002	0.98–0.99	0.99	.077	0.98–1.00
RWT	1.13	.411	0.85–1.50			
NT-proBNP/1000	1.04	.001	1.02–1.07	1.03	.049	1.00–1.07
LVEF	0.97	.002	0.96–0.99	0.98	.082	0.97–1.00
LVGLS	0.93	.009	0.88–0.98			

*Note:* IHD: ischaemic heart disease; eGFR: estimated glomerular filtration rate; LVEF: left ventricular ejection fraction; LVGLS: left ventricular global longitudinal strain.

## Discussion

In the present study, we demonstrated an increase in the number of newly diagnosed patients with ATTRwt from 2017 to 2022 with a trend towards a rise in the number of patients classified with NAC disease stage I. The diagnostic red flags identified were primarily cardiac-related ATTR signs, that is, heart failure in patients with increased left ventricular wall thickness, elevated NT-proBNP, impaired GLS with an apical sparing pattern and elevated troponins. Hospitalization due to WHF occurred in one-third of all patients, with the lowest WHF admission incidence in patients with NT-proBNP < 1000 ng/L. This patient group also had significantly lower risk of the combined endpoint WHF hospitalization or all-cause death. Finally, Cox regression analysis identified a prior implantation of PPM and NT-proBNP as independent predictors of WHF hospitalization with death as a competing risk.

During the last decade, several studies have reported ATTRwt as the underlying cause of heart failure with preserved ejection in older individuals or as part of other cardiac disease entities, such as aortic stenosis, conduction disturbances and hypertrophic cardiomyopathy, which have led to an increased awareness of ATTRwt [9]. However, limited data exist on the impact of diagnostic awareness of ATTRwt and the use of non-invasive diagnostic methods on the number of patients diagnosed and the changes in clinical characteristics and prognosis. Data from a global international longitudinal survey on ATTRwt demonstrated a fourfold increase in the number of diagnosed patients from 2012 to 2019 [7]. During the same timeframe, the proportion of patients classified in New York Heart Association (NYHA) class III-IV decreased from 46.4% in 2012 to 16% in 2019.

Conversely, there was an increase in patients classified as NYHA class II, rising from 35.7% to 47% during the same period suggesting earlier diagnosis of ATTRwt with less advanced disease in recent years. Temporal changes in patients diagnosed with ATTR have also been explored by Lane et al. who reported an exponential increase in ATTRwt from 2000 to 2017 [3]. In the present study, we report a similar increase in newly diagnosed patients; however, in a more recent period of investigation. The exact prevalence of ATTRwt is unknown, however, a recent study estimated the prevalence of ATTRwt in one out of 6000 in the general population based on retrospective heart failure data [18]. Given that these estimates can be confirmed by prospective data using uniform guideline-recommended diagnostic methods and along with increasing ageing in the general population, the cardiology community must be prepared for an additional increase in ATTRwt patients in the near future [14]. During the latter period of investigation (2020–2022), we noted an increase in the number of patients with NAC disease stage I at the time of diagnosis. Moreover, more than one-fifth of patients had NT-proBNP levels < 1000 ng/L, which is likely to have both clinical as well as prognostic implications. Recent data indicate that patients with stage I NAC disease with low NT-proBNP levels are characterized by lower NYHA class, lesser use of loop diuretics, better functional capacity, higher ejection fraction and a survival rate comparable to that of the general population [13]. This study contributes to the current prognostic knowledge by demonstrating that patients in NAC I and those with low NT-proBNP levels (< 1000 ng/L) had significantly lower risk of both WHF hospitalization and all-cause mortality.

Early diagnosis of patients with only mild disease is essential, thus enabling a greater yield of novel specific medical therapies, such as tafamidis, which has demonstrated pronounced beneficial effects in patients with less advanced ATTR disease [11]. To avoid diagnostic delay of ATTR in general, the use of several cardiac and extracardiac red flags have been recommended to raise suspicion of the disease [14]. To the best of our knowledge, the constellation of cardiac and extracardiac red flags in contemporary ATTRwt patients has not been previously reported. As expected, the finding of increased LV wall thickness was the main echocardiographic feature, but it is noteworthy that 4% of patients had normal LV wall thickness. The vast majority of reported red flags in our ATTRwt population were cardiac, emphasizing the importance of awareness to these signs and clinical scenarios for the timely diagnosis of ATTRwt among elderly patients. Thus, ATTR suspicion should be raised in cases of increased NT-proBNP, persistent troponin elevation, decreased LVGLS with an apical sparing pattern and the need for loop diuretics in patients with increased wall thickness especially those with normal to mildly reduced ejection fraction. Additional supportive signs include conduction disturbances, ECG changes such as pseudo-infarction or low-voltage pattern and aortic stenosis. In the present study, the only extracardiac red flag was previous carpal tunnel syndrome surgery, which is consistent with previous studies [13,19,20]. The distribution of red flags in the two time periods was comparable, except for that of troponin I and LVGLS with apical sparing pattern. Troponin I level was significantly lower in the latter time period, suggesting less advanced disease.

Recent data reported a median survival time of 57 months in ATTRwt patients [3] and a median survival of 86% with a median follow-up time of 21 months in NAC disease stage I patients [3].

In a recent retrospective study, the characteristics of contemporary pathways leading to ATTRwt diagnosis were described in 1281 patients diagnosed between 2016 and 2021 [21]. Overall survival was 77% in all patients, with a median of 47 months follow-up which seems more favourable than our cohort with a survival of 69% with a median of 26 months follow-up. This difference may be attributed to age differences as our patients were older (82 vs. 78 years). However, contrasting our study, no data with respect to WHF hospitalization, echocardiographic data or biomarkers were presented. In our patients with NAC disease stage I, the two-year survival rate was 91%. Even better survival was found in patients with NT-proBNP < 1000 ng/L, demonstrating a two-year survival of 95%.

Hospitalization due to WHF is another important event commonly observed in ATTRwt patients, both preceding and following diagnosis establishment [3,22]. A recent study reported ATTR patients using hospital services a median (IQR) of 17 (9–27) times during the three years prior to diagnosis [3]. WHF hospitalization reduces quality of life, leads to significantly higher health-related costs and is associated with increased mortality in ATTRwt patients [3,22–24]. In the present study, at least one hospitalization due to WHF occurred in 27% of patients during the first year and in 48% of patients within three years after diagnosis. Risk of hospitalization due to WHF was low in patients with NT-proBNP < 1000 ng/L (13% after one year), whereas it was almost three times as high in patients with NT-proBNP ≥ 3000 ng/L (37% after one year). So far, no prognostic information with respect to WHF hospitalization has previously been reported. In this study, we observed a significantly lower rate of WHF hospitalization or death in both patients categorized as NAC I and those with NT-proBNP levels below 1000 ng/L. These findings are encouraging, in addition to the fact that we observed an increase in patients diagnosed in NAC disease stage I during the last three years as compared to the first three years (58% vs. 49%). New specific medical treatments with different modes of action are currently being investigated, and recently acoramidis, a transthyretin stabilizer, demonstrated significant effect on morbidity and mortality in ATTR patients [25]. Despite observing a higher number of patients in NAC I during the later period, we were unable to detect any differences in echocardiographic structural and functional parameters between the two time periods. Recent findings suggest that monitoring changes in NT-proBNP levels and diuretic dosage may be the most optimal approach for assessing disease stage and progression, surpassing the utility of other clinical or echocardiographic variables [26].

In our study, aortic stenosis was an independent predictor of WHF hospitalization. Previous studies have drawn attention to the fact that cardiac amyloidosis and aortic stenosis coexist more frequently than previously suspected with a prevalence ranging from 6% to 16% [27]. Evaluation of aortic stenosis severity in ATTRwt patients can be challenging due to low stroke volume and lower transvalvular gradients and can easily be evaluated as mild or moderate despite the presence of a more severe aortic stenosis. Misclassification of aortic stenosis severity may cause insufficient treatment and most likely increase the risk of hospitalization with WHF. Recent data on ATTR patients with severe aortic stenosis reported worse prognosis if not treated by transcatheter aortic valve replacement (TAVR) and with the same mortality rate as non-ATTR patients if TAVR was performed [28].

Prior implantation of PPM was found to be an independent predictor of WHF hospitalization in the present study, as well as in a previous study by our group [22]. In the study by Ladefoged et al. patients with PM at baseline had significantly higher NT-proBNP, lower eGFR, higher LV mass index, reduced LV and RV systolic function, compared with patients without PM, suggesting more advanced disease in PM patients. Increasing data support that a considerable proportion of patients with cardiac amyloidosis develop conduction system disturbances that require PM implantation. In a recent study of 405 patients with cardiac amyloidosis (29.4% AL, 14.6% variant ATTR and 56% ATTRwt), 8.9% received a PM within three years of diagnosis [29].

In recent guidelines, treatment with SGLT2i was recommended in heart failure patients regardless of EF to reduce the risk of HF hospitalizations or cardiovascular death [30]. In a recent propensity-matched comparison of patients with ATTR cardiomyopathy [31], treatment with SGLT2i was well tolerated and associated with favourable effects on heart failure symptoms. SGLT2i treatment was associated with reduced risk of HF hospitalization and cardiovascular and all-cause mortality, regardless of the ejection fraction. As stated by the authors, these results should be confirmed in randomized controlled trials. The study design and number of matched patients could not account for unmeasured confounders, indication bias of SGLT2i initiation and changes of covariates during the study (including the introduction of specific ATTR treatment such as acoramidis [25]. However, the data from Porcari et al. is encouraging. Combined with the findings showing the risk of HF hospitalization can be predicted by loop diuretic dosage and NT-proBNP levels, this study may help clinicians identify which ATTR patients could benefit the most from SGLT2i treatment.

Our study was a single-centre study, which limits the interpretation of data from a general perspective. We reported all-cause mortality and not the specific cause of death, which could have strengthened our data. However, previous data from our institution showed that the main cause of death in ATTRwt was heart failure [22]. In this prospective study, the median follow-up time was 26 months, which only provides short-to mid-term prognosis, but seems comparable to other large prospective studies in ATTRwt patients. Nearly one-third of the included ATTRwt patients were enrolled in ongoing randomized placebo-controlled trials testing specific ATTR treatments, which may influence the prognostic results presented. Finally, our dataset was not fully complete regarding troponin as our institution has used three different troponin assays during the study period, resulting in short periods of incomplete troponin measurements in out clinic patients.

## Conclusion

In this study of contemporary ATTRwt patients, we noted a 37% increase in the number of newly diagnosed patients in 2020–2022 as compared with 2017–2019 in parallel with tendency towards a greater proportion of patients presenting with less advanced disease in terms of NAC disease classification at the time of diagnosis. Cardiac red flags dominated the clinical scenario in particular elevation of NT-proBNP, troponins, reduced LVGLS with apical sparing pattern and heart failure related with loop diuretics. Risk of WHF hospitalization or death was significantly lower in patients with NAC disease stage I or in patients with NT-proBNP <1000 ng/L. Independent predictors of WHF hospitalization or death were NT-proBNP and prior implantation of PPM. Our findings underline the importance of continuous attention to diagnostic awareness, as early diagnosis is critical to initiate both general and specific ATTR treatment, thus improving prognosis.

## Data Availability

Raw data were generated at Aarhus University Hospital. The derived data supporting the findings of this study are available from the corresponding author [SBL] on request.
